# Prognostic and diagnostic significance of galectins in pancreatic cancer: a systematic review and meta-analysis

**DOI:** 10.1186/s12935-019-1025-5

**Published:** 2019-11-21

**Authors:** Qiqing Sun, Yiyin Zhang, Mengqi Liu, Zeng Ye, Xianjun Yu, Xiaowu Xu, Yi Qin

**Affiliations:** 1 Department of Pancreatic Surgery, Fudan University Shanghai Cancer Center, Shanghai, 200032 China; 2 Department of Oncology, Shanghai Medical College, Fudan University, Shanghai, 200032 China; 3 Pancreatic Cancer Institute, Fudan University, Shanghai, 200032 China; 4 Shanghai Pancreatic Cancer Institute, Shanghai, 200032 China

**Keywords:** Galectins, Pancreatic cancer, Prognosis, Diagnosis, Meta-analysis

## Abstract

**Background:**

Galectins constitute a family of β-galactoside-binding proteins, which influence various hallmarks of pancreatic cancer, including cell proliferation, invasion and migration; immune escape; and angiogenesis. Although many studies have concentrated on the role of galectins in pancreatic cancer, the results remain controversial. Hence, we performed a comprehensive meta-analysis to clarify the precise diagnostic and prognostic value of galectins in pancreatic cancer.

**Methods:**

PubMed/MEDLINE, EMBASE and Web of Science were used to search related published literature up to July 2019. Pooled hazard ratios (HRs), diagnostic accuracy variables and related 95% confidence intervals (CIs) were calculated using STATA 14.0 software.

**Results:**

Eleven studies including 1227 participants met our inclusion criteria. High expression of galectin family was not correlated with overall survival (OS) in pancreatic cancer (HR, 1.19; 95% CI 0.67–2.11). According to subgroup analysis, high levels of galectin-1 were significantly correlated with worse OS in pancreatic cancer (HR, 4.77; 95% CI 2.47–9.21), while high levels of tandem-repeat galectins (galectin-4 or galectin-9) predicted both better OS (HR, 0.63; 95% CI 0.46–0.86) and disease-free survival (DFS) (HR, 0.63; 95% CI 0.48–0.83). The expression levels of galectin-3 did not directly correlate with prognosis (HR, 0.99; 95% CI 0.40–2.46). The pooled sensitivity, specificity, positive likelihood ratio, and negative likelihood ratios of galectin-3 were 0.64 (95% CI 0.41–0.82), 0.76 (95% CI 0.59–0.88), 2.70 (95% CI 1.21–6.1), and 0.47 (95% CI 0.23–0.98), respectively. The area under the curve (AUC) of galectin-3 was 0.77.

**Conclusion:**

Taken together, our results suggest that high expression of galectin-1 and low levels of galectin-4 or galectin-9 are predictors of worse prognosis in pancreatic cancer patients. The expression of galectin-3 was not directly related to OS and other clinical characteristics. Although galectin-3 exhibited some diagnostic value in patients with pancreatic cancer in this meta-analysis, clinical application prospects remain to be validated. Further studies are warranted to confirm and strengthen these findings.

## Background

Pancreatic cancer is among the most aggressive malignancies due to limited early diagnosis and therapeutic strategies, ranking as the fourth leading cause of cancer-related deaths [1, 2]. Despite enormous advances in diagnostic and therapeutic strategies made in many other tumours over the past decades, the prognosis of pancreatic cancer remains unsatisfactory, with a 5-year survival rate of approximately 7% [3, 4]. Most patients with pancreatic cancer are diagnosed at advanced stages, and a lack of effective biomarkers for identifying patients with a high risk for recurrence contributes to poor prognosis.

Galectins constitute a family of multifunctional proteins that share similar β-galactoside-binding affinity and carbohydrate-recognition domains (CRDs) [5]. Galectins are divided into three subtypes (prototype, chimeric and tandem-repeat) based on their structural differences [6]. Prototype galectins (galectin-1, 2, 5, 7, 10, 11, 13, 14 and 15) contain one CRD that can homodimerize. Tandem repeat galectins (galectin-4, 6, 8, 9 and 12) consist of two CRDs that are connected by a linker up to 70 amino acids in length. Chimeric galectin (galectin-3) consists of a single CRD fused to unusual tandem repeats of proline- and glycine-rich short stretches (a total of approximately 120 amino acids) [7]. Eleven galectins have been identified in humans, among which, galectin-1, 3 and 9 have been the most widely studied across different fields [8]. Galectins can be localized both inside and outside of cells. Secreted galectins can crosslink with cell-surface glycoconjugates covered with galactose-containing oligosaccharides to induce intracellular signaling, including mitosis, apoptosis and cell-cycle progression. Intracellular galectins shuttle between the cytoplasm and nucleus to participate in processes such as pre-mRNA splicing [7]. In this review, we will discuss four different galectins. Galectin-1 (LGALS1, 22q13.1) belongs to the prototype galectins, and is usually released from stromal cells and endothelial cells [9]. Galectin-3 (LGALS3, 14q22.3) is the only galectin that belongs to the chimeric galectin, and was first described to be localized to the outer membrane of macrophages (Mac-2 antigen) [10]. It is also found in endothelial cells, immune cells and fibroblasts, and can be transported from the nucleus to the cytoplasm to interact with mitochondria and regulate apoptosis [11, 12]. Galectin-4 (LGALS4, 19q13.2) and galectin-9 (LGALS9, 17q11.2) belong to the tandem repeat type galectins and are involved in inflammatory and immune processes [13, 14]. Recent studies have elucidated the biological functions of galectins in tumors, including in the regulation of oncogenesis, cancer cell growth, apoptosis, cell adhesion, migration and immune escape [8].

Evidence has suggested that altered expression of galectins in pancreatic cancer tissues may have a role in tumour carcinogenesis, proliferation, progression, angiogenesis, metastasis and immune response [15–18]. Recent studies have also raised the possibility of using galectins in diagnostic, prognostic and other clinical characteristics, such as tumour node metastasis (TNM) stage pathological grade [17, 19–28]. Although many studies have concentrated on the correlation between galectins and pancreatic cancer, conclusions remain controversial. Therefore, we performed a comprehensive meta-analysis to clarify the diagnostic and prognostic role of galectins in pancreatic cancer.

## Methods

### Literature search

We undertook a comprehensive and systematic review by searching following databases, PubMed/MEDLINE, EMBASE and Web of Science. The species were limited to human, and the language was limited to English. The retrieval strategy was listed as follow: (Gal OR galectin OR S-type lectin OR galactose binding lectin OR D galactoside binding lectin OR galaptin OR beta galactoside binding lectin OR half curling element) AND (pancreatic OR pancreas) AND (cancer OR tumour OR tumor OR carcinoma Or adenocarcinoma OR neoplasia OR neoplasm) AND (prognosis OR prognostic OR prognoses OR prognos OR predict OR survival OR outcome OR biomarker OR diagnosis OR diagnostic). Reference lists of literature were manually retrieved for additional information. Studies included in this review are from peer reviewed journals. Two reviewers (Sun QQ and Zhang YY) independently scrutinized the initially identified articles for the assessment of eligibility. The last search time was in June 2019.

### Selection criteria

The following inclusion criteria had to be fulfilled: (1) clinical studies of patients with pancreatic cancer; (2) a diagnosis of pancreatic cancer was confirmed by histopathology; (3) explicit methods for the detection of galectins in patients; (4) the cut-off value of galectin expression level was described; (5) studies described the correlation between galectin expression and survival outcome or diagnostic value, and for survival outcome, hazard ratio (HR) values and corresponding 95% confidence intervals (CIs) for overall survival (OS) or disease free survival (DFS) were either described in the studies or could be calculated by the published data; (6) the most recent or the most integrated report would be included when study populations overlapped. Exclusion criteria were as follows: (1) experiments not based in patients; (2) duplicated studies; (3) literature published as abstracts, letters, reviews, case reports, editorials and expert opinions; (4) no full text available or unable to extract the outcome indicators; (5) non-English publications. Any disagreement between the two reviewers was resolved by discussion or consultation with a third reviewer (Liu MQ).

### Data extraction

The information extracted from each article were listed as follows: (1) baseline characteristics including first author’s name, publication year, region, sample size, gender and age of the patients, galectin type, TNM stage, treatment and the follow-up duration; (2) method to determine the expression level of galectins and cut-off value; (3) clinical outcomes including OS or DFS and HRs and its 95% CI. If an article only had Kaplan–Meier curves, we extracted survival data from the curves indirectly by Engauge Digitizer software before we put the data into a spreadsheet, called Tierney table to estimate its correlative HRs with 95% CI [29]; (4) diagnostic values including the number of true positives (TPs), false positives (FPs), true negatives (TNs); false negatives (FNs).

### Quality assessment

Two reviewers (Sun QQ and Zhang YY) systematically assessed the methodological quality of each study independently using the Newcastle–Ottawa Quality Assessment Scale (NOS) [30], which is primarily used in non-randomized studies. A study with NOS > 5 was regarded as a high-quality study [31]. Additionally, the quality assessment approach reported by Hayden et al. was used to assess the quality of prognostic studies. This scale contains six aspects of measurement, including study participation, study attrition, prognostic factor measurement, outcome measurement, confounding measurement and account, and analysis [32]. Quality Assessment of Studies of Diagnostic Accuracy included in Systematic Reviews (QUADAS-2) was used to assess the methodological quality of diagnosis in this review [33]. Four key domains are included in this criteria: patient selection, index test, reference standard, and flow and timing. Divergence was resolved under discussion or consultation.

### Statistics analysis

Hazard ratios (HRs) were used to assess survival data, while odds ratios (ORs) were chosen for dichotomous data with corresponding 95% confidence intervals (CIs). Heterogeneity among studies was evaluated using Cochran’s Q test and Higgins *I*-squared statistics. The random-effects model was applied under obvious heterogeneity (*I*^2^ > 50% and/or *P* < 0.1); otherwise, the Mantel–Haenszel fixed-effects model was applied [34]. The pooled sensitivity, specificity, positive likelihood ratio (PLR), negative likelihood ratio (NLR), and diagnostic odds ratio (DOR) were calculated with corresponding 95% CIs. The summary receiver operating characteristic curve (SROC), were analysed according to the statistical methods described in a previous study [35]. We used area under the curve (AUC) to summarize diagnostic values. Potential publication bias of prognosis was assessed using Begg’s funnel plot and Egger’s test [36], and bias of diagnosis was evaluated using Deeks’ funnel plot [37]. The potential subgroup analysis was conducted considering the galectin subtype, region and sample size on OS and DFS. All analyses were conducted using STATA version 14.0 (Stata Corporation, College Station, TX, USA) and Review Manager (RevMan) version 5.3. (Cochrane Collaboration, Oxford, UK), and *P *< 0.05 was considered statistically significant.

## Results

### Characteristics of included studies

**Fig. 1:**
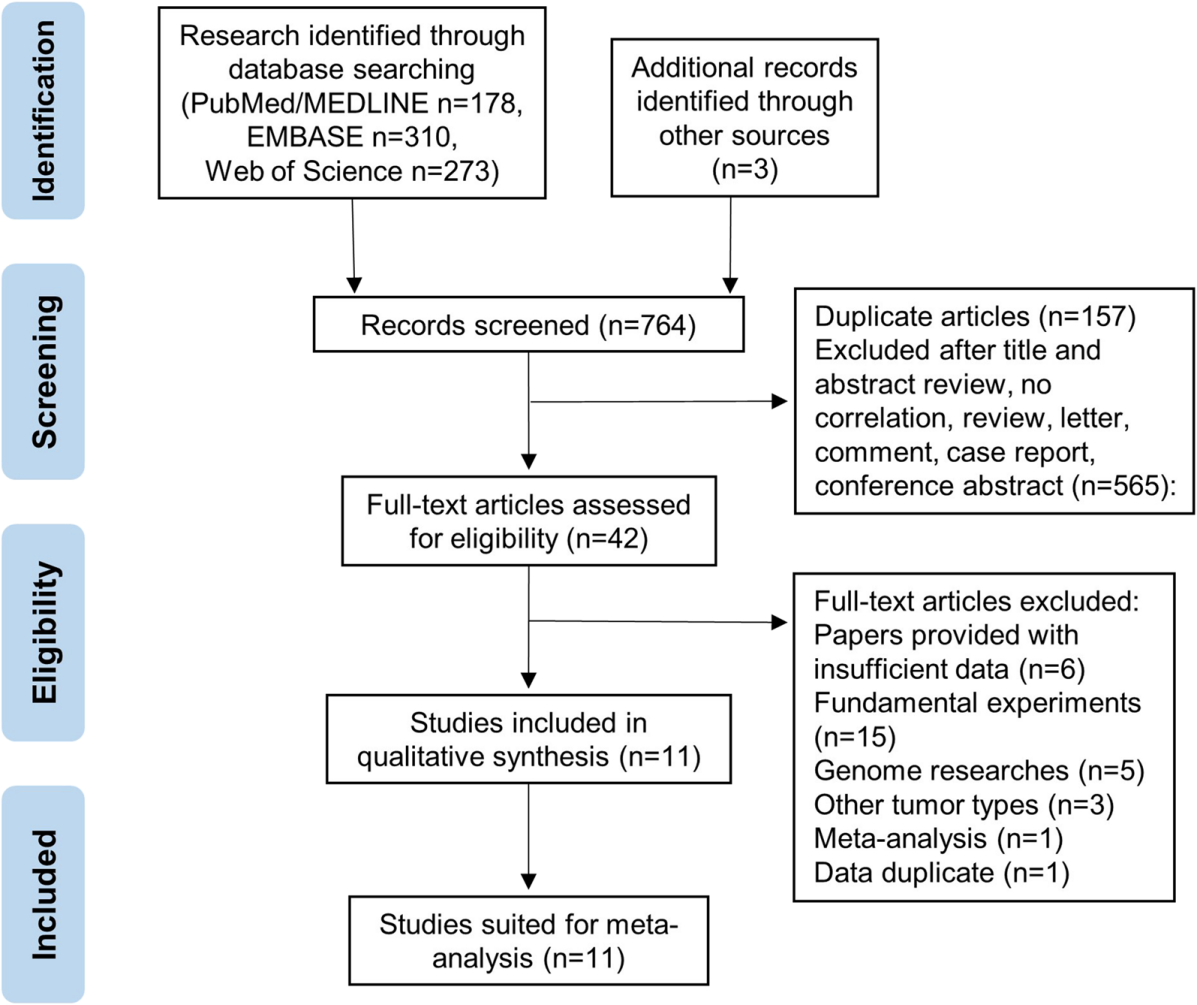
Literature review process

The flow diagram for article selection is illustrated in Fig. 1. We initially retrieved 761 publications from the four databases. 178 articles came from Pubmed/MEDLINE, 310 articles came from EMBASE, and 273 articles came from Web of Science. Then, we added 3 studies identified through other sources. After manually screening titles, abstracts, and keywords and removing duplicates, 42 studies remained for further full-text article assessment. Thirty-one publications were further excluded, among which six papers were excluded due to insufficient data, fifteen papers were fundamental experiments, five were genome research, three were found to be other tumor types, one were meta-analysis and one paper were discovered to be data duplicate. Ultimately, 11 studies comprising 1227 participants were eligible for the meta-analysis on the association between galectins and pancreatic cancer, and detailed information concerning these studies included in prognostic and diagnostic part is summarized in Tables 1 and 2 repectively. All studies were retrospective with publication years ranging from 2002 to 2019. Eight studies with 672 participants were enrolled for prognostic analysis, and all HRs and correlated 95% CIs were obtained using the methods mentioned above. Participants in this meta-analysis were from America, China, France, Germany, Japan, Italy, Netherlands, Sweden, and Taiwan. The cut-off values for galectins varied among the different studies. All pancreatic cancer patients’ diagnoses were confirmed by pathological examination.

**Table 1:** Summary of the prognosis part of included studies

Study	Region	Galectin types	Sample size	High expression (%)	Age (mean or median)	Sex (M/F)	Pathology	Stage or grade	Result	Survival analysis	HR (95% CI)	Cutoff value	Sample source	Detection method	Follow-up (months)
Chen [19]	USA	Galactin-1	43	95	NR	NR	PDAC	NR	OS	U	4.90 (1.788–13.426)	Positive cells ≥ 5%	Tissue	IHC	72
Tang [17]	China	Galactin-1	66	71.21	55 (37–83)	45/21	PDAC	TNM I-IV	OS	M	4.676 (1.963–11.134)	Positive cells > 30%	Tissue	IHC	78
Shimamura [20]	Japan	Galactin-3	104	50	62 (45–82)	62/42	PDAC	TNM I-IV	OS	M	0.48 (0.28–0.81)	Positive cells ≥ 60%	Tissue	IHC	104
Gaida [21]	Germany	Galactin-3	130	80.80	66 (39–85)	74/56	PDAC	TNM I-IV	OS	KM	0.85 (0.49–1.47)	Positive cells ≥ 1%	Tissue	IHC	40
Shimura [24]	Japan	Galactin-3	21	47.62	67.1	14/7	PC	TNM I-III	OS	M	4.559 (1.176–17.685)	10.2 ng/ml	Serum	Immunoassay	66
Maftouh [26]	Italy	Galactin-4	20	55.00	NR	9/11	PDAC	T3N0(1)Mx	OS	KM	0.64 (0.25–1.68)	Staining score ≥ median	Tissue	IHC	45
									DFS	KM	0.87 (0.33–2.26)				
Hu [27]	Sweden	Galactin-4	140	79.30	69 (63–73)	66/74	PDAC	TNM I–IV	OS	M	0.636 (0.380–1.063)	Positive cells ≥ 10%	Tissue	IHC	60
									DFS	M	0.638 (0.371–1.095)				
Sideras [28]	Netherlands	Galactin-9	148	53.30	NR	NR	PC	Grade I-III	OS	M	0.62 (0.40–0.97)	0.4 (out of 3)	Tissue	IHC	175
									DFS	U	0.6 (0.43–0.85)				

*DFS* disease-free survival, *HR* hazard ratio, *IHC* immunohistochemistry, *KM* Kaplan–Meier analysis, *M/F* male/female, *M* multivariate analysis, *NR* not reported, *OS* overall survival, *PC* pancreatic cancer, *PDAC* pancreatic ductal adenocarcinoma, *U* univariate analysis

**Table 2:** Characteristics of the diagnosis part of included studies

Study	Region	Galectin type	Patient					Control			TP	FP	FN	TN	Cutoff value	Sample source	Detection method
			Number	Age (M)	Sex (M/F)	Pathology	Stage	Number	Age (M)	Sex (M/F)							
Xie [22]	China	Galactin-3	49	NR	NR	PDAC	TNM I–IV	88	NR	NR	37	8	12	80	3.77 ng/ml	Serum	Immunoassay
Coppin [23]	France	Galactin-3	44	64 (41–82)	22/22	PDA	TNM I–IV	58	60 (41–89)	42/16	12	25	32	33	23.6 ng/ml (male), 27.2 ng/ml (female)	Serum	Immunoassay
Shimura [24]	Japan	Galactin-3	21	67	14/7	PC	TNM I-III	35	55 (25–84)	20/15	18	14	3	21	6.2 ng/ml	Serum	Immunoassay
Liao [25]	Taiwan	Galactin-3	91	63	58/33	PC	TNM I–IV	91	59	70/21	61	16	30	75	6.5 ng/ml	Serum	Immunoassay

### Quality assessment

According to NOS, results for the assessment of methodological quality of prognosis are reported in Additional file 1: Table S1, with all studies achieving a score greater than 5. According to the QUADAS-2 analysis, results of the quality assessment of studies included in the diagnostic analysis are shown in Additional file 2: Fig. S1.

### Meta-analysis results of prognostic significance

**Fig. 2:**
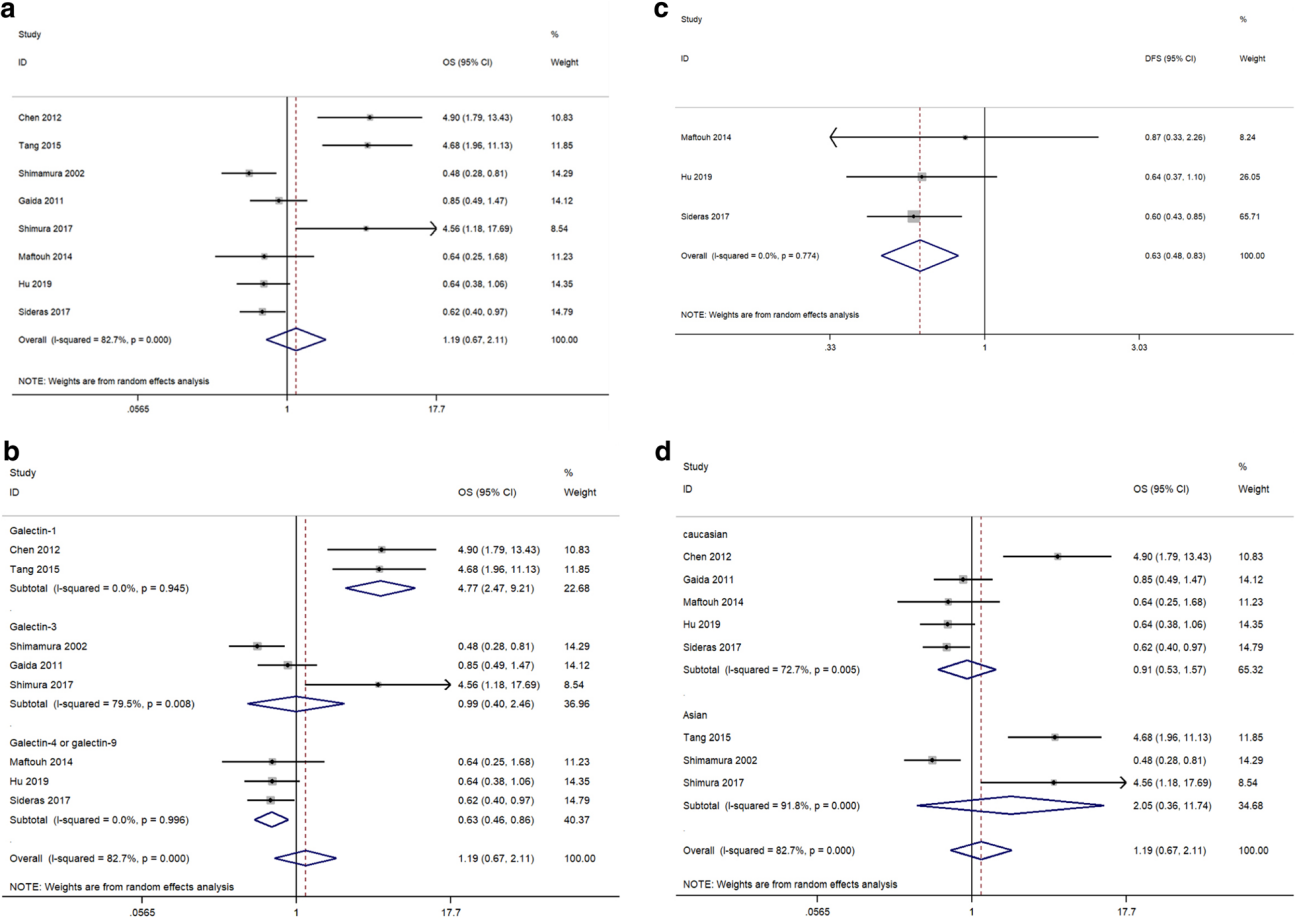
Forest plots of OS or DFS in association with galectins in pancreatic cancer. **a** The overall group. **b** The subgroup analysis of galectin types on OS. **c** The subgroup analysis of galectin types on DFS. **d** The subgroup analysis of dominant ethnicity

A total of 8 studies were included in the pooled survival analysis of OS. As described in Fig. 2a, results indicated that the galectin family is not correlated with OS in pancreatic cancer (HR, 1.19; 95% CI 0.67–2.11). Due to significant heterogeneity (*I*^2^ = 82.7%, p < 0.001), the random-effects model was applied to the analysis, and subgroup analysis were applied to seek for causes of heterogeneity. In the subgroup analysis by subtype of galectins, two studies were conducted in prototype galectins (two of galectin-1), three studies were in chimeric galectins (three of galectin-3), and three studies were in tandem-repeat galectins (two of galectin-4, one of galectin-9). In addition, survival analysis of DFS was conducted in the subgroup of tandem-repeat galectins. The heterogeneity (*I*^2^) decreased from 82.7% to 0%–79.5% after subgroup analysis of different galectin subtypes, which indicated that different galectin subtype is among the main cause of heterogeneity. High levels of galectin-1 were significantly correlated with poor OS in pancreatic cancer (HR, 4.77; 95% CI 2.47–9.21, Fig. 2b). Galectin-3 was not correlated with OS in pancreatic cancer (HR, 0.99; 95% CI 0.40–2.46, Fig. 2b). Conversely, high levels of tandem-repeat galectins (galectin-4 or galectin-9) predicted both better OS (HR, 0.63; 95% CI 0.46–0.86, Fig. 2b) and DFS (HR, 0.63; 95% CI 0.48–0.83, Fig. 2c) in pancreatic cancer. In addition, in the subgroup analysis by region, five studies were in Caucasian populations, and three studies were in Asian populations. The significant heterogeneity in different subgroups (Caucasian: HR, 0.91; 95% CI 0.53–1.57, *I*^2^ = 72.7%; Asian: HR, 2.05; 95% CI 0.36–11.74, *I*^2^ = 91.8%, Fig. 2d) indicated that different regions may not be among the main cause of heterogeneity.

### Meta-analysis results of diagnostic value

**Fig. 3:**
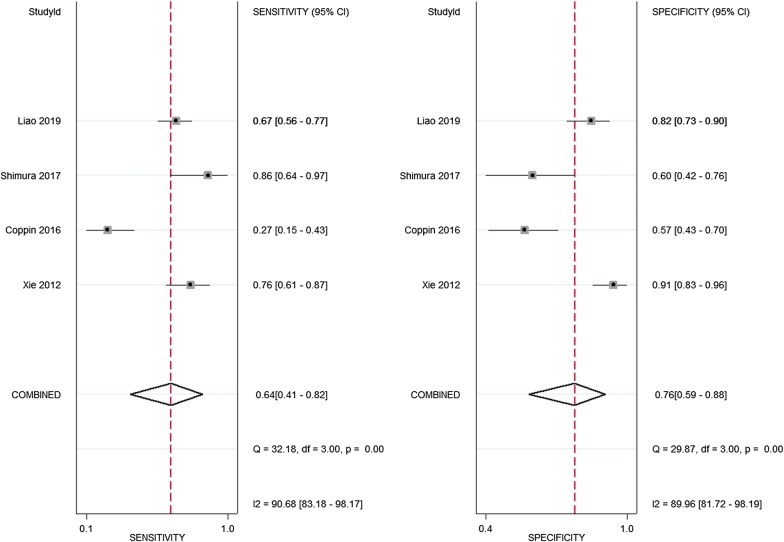
Forest plots of sensitivity and specificity of galectin-3 in pancreatic cancer

The characteristics of the studies included for diagnostic analysis are summarized in Table 2. The diagnostic accuracy of galectin-3 was evaluated. The pooled sensitivity of serum galectin-3 was 0.64 (95% CI 0.41–0.82), specificity was 0.76 (95% CI 0.59–0.88), PLR was 2.70 (95% CI 1.21–6.1), and NLR was 0.47 (95% CI 0.23–0.98) (Fig. 3). DOR combines the strengths of sensitivity and specificity, being a measure of the effectiveness of a diagnostic test. The pooled DOR of galectin-3 was 5.93 (95% CI 0.96–36.72, Additional file 2: Fig. S2a). The heterogeneity of these studies was assessed by *I*^2^ values of the diagnostic variables. The random-effects model was applied due to the substantial heterogeneity among the studies (*I*^2^ for sensitivity 90.68%, *p *< 0.01; *I*^2^ for specificity 89.06, *p *< 0.001; *I*^2^ for DOR 90.68%, *p *< 0.001). The SROC curve was applied to represent the accuracy of diagnostic testing by combining sensitivity and specificity, with an AUC approaching one reflecting a well-performed and precise diagnosis [38]. In this meta-analysis, the AUC of galectin-3 was 0.77 (95% CI 0.74–0.80, Additional file 2: Fig. S2b).

### Meta-analysis results of for clinical characteristics

We then next undertook a meta-analysis concerning the cooperation between the expression of galectins and clinical characteristics of pancreatic cancer (Table 3). As shown in the table, neither high expression of galectins nor galectin-3 in pancreatic cancer were associated with clinical characteristics.

**Table 3:** Correlation of galectins with clinical characteristics

Clinical characteristics	Galectins				Galectin-3			
	No. of studies	OR	95% CI	*I*^2^ (*P*)	No. of studies	OR	95% CI	*I*^2^ (*P*)
TNM stages III + IV vs. I + II	5	0.529	0.252–1.109	13.1% (0.327)	3	0.364	0.106–1.244	21.1% (0.281)
Invasion depth T3 + T4 vs. T2 + T1	6	1.476	0.366–5.947	75.6% (0.003)	3	0.870	0.091–8.301	65.3% (0.056)
Perineural invasion positive vs. negative	4	1.302	0.735–2.304	0.0% (0.415)	1	1.226	0.350–4.299	–
Vascular invasion positive vs. negative	2	2.135	0.514–8.857	50.0% (0.157)	1	1.295	0.573–2.929	–
Lymphatic invasion positive vs. negative	6	0.902	0.501–1.626	29.4% (0.215)	4	0.722	0.380–1.373	0.0% (0.594)
Distant metastasis positive vs. negative	3	0.837	0.397–1.768	0.0% (0.611)	3	0.837	0.397–1.768	0.0% (0.611)
Differentiation grade poor + vs. well + moderate	5	0.791	0.256–2.442	75.2% (0.003)	2	1.066	0.506–2.243	0.0% (0.859)

### Sensitivity analysis and publication bias

**Fig. 4:**
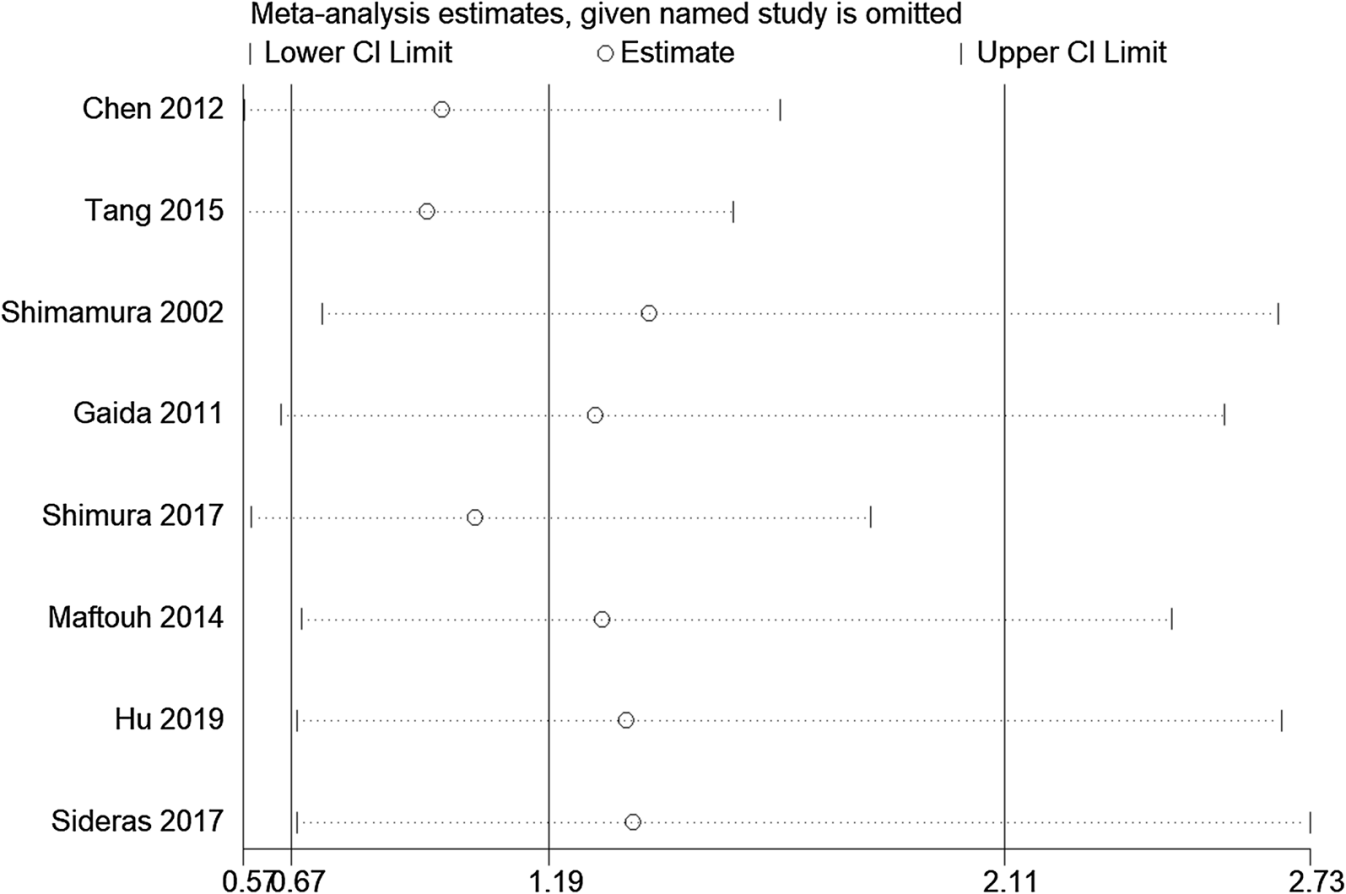
Sensitivity analysis of prognosis from the included study

Sensitivity analysis was applied to detect the stability of the pooled HR and its 95% CI, and results indicated that no significant heterogeneity was present among the included studies (Fig. 4). Begg’s funnel plot and Egger’s test were applied to evaluate the publication bias for OS. Begg’s tests showed that publication bias was not significant for the enrolled studies (*p *= 0.108, Additional file 2: Fig. S3a). However, Egger’s test revealed that publication bias did exist among the studies (*p *= 0.022). Additionally, Deeks’ tests suggested that there was no significant publication bias for the diagnostic analysis (*p *= 0.77, Additional file 2: Fig. S3b).

## Discussion

Due to the lack of early diagnosis and the poor survival rate after surgery, efforts have been made to identify novel diagnostic and prognostic biomarkers in patients with pancreatic cancer. Carbohydrate antigen 19-9 (CA19-9) is the most widely used serum biomarker for detecting pancreatic cancer. The correlation between CA19-9 and surgical outcomes has been described in many studies and indicates that CA19-9 could assist in identifying resectable or nonresectable pancreatic cancer. However, the thresholds identified in each study were varied [39]. Our team previously conducted a series of relevant studies in this field [40–42]. We found that the serum signature of CEA^+^/CA125^+^/CA19-9 > 1000 U/ml was a preoperative indicator for worse surgical outcome in pancreatic cancer, even though the R0-resection was successfully conducted. However, no consensus has been reached on how CA19-9 serum levels change and their predictive value in managing pancreatic cancer patients. A pooled analysis of 6 prospective trials indicated that baseline CA19-9 is prognostic in patients with advanced pancreatic cancer who underwent treatment with gemcitabine-containing regimens. However, reduced CA19-9 after the second cycle of chemotherapy is no longer predictive [43]. Therefore, multiple studies have been conducted to search for novel biomarkers that provide earlier or more accurate prediction for pancreatic cancer.

As part of the lectin superfamily, galectins are soluble proteins widely expressed in a variety of cells, exerting their primary biological functions both intracellularly and extracellularly [44]. In general, galectins are involved in diverse biological process, including regulation of cell signalling, progression of the cell cycle, apoptosis, pre-mRNA splicing and cell motility and adhesion [45, 46]. Consistent with these various biological functions, altered expression levels or dysfunction of galectins have been correlated with the development of diseases, such as cancer. Evidence has suggested the influence of galectins on different hallmarks of pancreatic cancer, including cell proliferation, invasion and migration, immune escape and angiogenesis [17, 47–49]. As a result, therapeutic strategies have been developed that target galectins in pancreatic cancer. Yao et al. suggested that HH1-1, a novel galectin-3 inhibitor, inhibited the progression of pancreatic cancer both in vitro and in vivo [49]. Shih et al. found that combination treatment of paclitaxel and LLS2, a novel galectin-1 inhibitor, enhanced toxicity in human pancreatic cancer cell lines [50]. However, for clinical applications, most clinical trials on galectin inhibitors combined with chemotherapy for the treatment of different tumors were neither withdrawn nor terminated, and only one trial has completed although no results are currently available (NCT00054977). Thus, the use of galectin inhibitors for the treatment of cancer has received renewed interest. Recently, a novel biomarker on the galectin-9 binding partner, T cell immunoglobulin mucin-3 (TIM-3), was found to be upregulated in response to anti-PD-1 therapy and has been targeted as a novel immune checkpoint in tumor immunotherapy [51]. Although these studies are still in their early stages, anti-TIM-3 agents (e.g., TSR-022, LY3321367) combining anti-PD-1/PD-L1 have been applied in several ongoing clinical trials (NCT02817633; NCT03099109), highlighting their promising role for the treatment of advanced solid tumors.

Many studies have also explored the prognostic role of galectins in cancer. Galectins are thought to be associated with patient outcome. Increasing evidence has suggested that galectin-1 is elevated in cancer tissues and a high expression level of galectin-1 is associated with poor OS and DFS in different cancer types, particularly in digestive cancers [19, 52]. Galectin-1 has also been proved to play oncogenic role by some researches in pancreatic cancer. Galectin-1 could regulate acinar-to-ductal metaplasia by promoting Hedgehog pathway signaling in PDAC cells and tumor-stroma crosstalk [16]. Galectin-1 also reportedly enhances the production of stromal cell-derived factor-1via NF-κB signalling, resulting in increased metastasis in pancreatic cancer both in vitro and in vivo [53]. In addition, a novel galectin-1 inhibitor LLS2 was found to potentiate the antitumor effects of paclitaxel in several human cancer cell lines including pancreatic cancer cells in vitro [50]. Consistent with the oncogenic role in pancreatic carcinogenesis, our results indicated that in patients with pancreatic cancer, high levels of galectin-1 were significantly correlated with poor OS, as complements to some previous meta-analysis in solid tumors, demonstrating that higher expression of galectin-1 was associated with worse prognosis in cancers [52], though more excellently-designed large-sized prospective researches are needed in the future.

However, another subtype of galectins, tandem-repeat galectins, seemed to exhibit the opposite picture in prognostic value. Although only limited tumor types were evaluated, higher galectin-9 expression was reported in a meta-analysis to be related to better prognosis in solid tumors especially in digestive cancers [54]. Due to the limited number of studies about galectin-4 and galectin-9, their biological behaviours and underlying mechanisms in malignancies still remain controversy. Galectin-9 was found to suppress the proliferation of pancreatic cancer cell lines, and metastatic liver cancer cell lines [55, 56]. Conversely, higher serum galectin-9 was observed in PDAC patients [57]. Inhibition of galectin-9 leads to significant tumour regression in a mouse model. Mechanistically, dectin-1 binds to galectin-9, resulting in immunogenic or tolerogenic phenotypes of CD4 + and CD8 + T cells that promote tumour progression in pancreatic cancer [58]. Though only a small number of studies have been performed, low expression of galactin-4 has been described to be significantly correlated with early recurrence and poor survival of pancreatic cancer [27]. Reduced expression of galectin-4 was also described in colorectal cancer, skin cancer and prostate cancer [59–62]. Our meta-analysis supported the various function of different galectin subtypes in cancer prognosis, that converse to galectin-1, high levels of galectin-4 or galectin-9 predicted better OS and DFS in pancreatic cancer.

The prognostic role of galectin-3 has been widely studied, but appears to be unclear and disparate between different cancer types. For example, although galectin-3 has been proven to be related to poor survival and play an oncogenic role in many types of cancer, such as ovarian, colorectal, and non-small cell lung cancer [63], high expression of galectin-3 appears to better predict survival in patients with gastric cancer [64]. Alterations in galectin-3 expression have also been reported in previous studies wherein it is implicated in cell proliferation, apoptosis, adhesion, and angiogenesis [65]. Therefore, substantial studies have explored its role in pancreatic cancer, and most studies proved evidence to support its oncogenic role. Overexpressed galectin-3 in pancreatic cancer cells induced cell proliferation and invasion by activating Ras signaling [15]. Silencing of galectin-3 decreased pancreatic cancer cell proliferation and cyclin-D1 levels [66]. Zhao et al. found that inhibition of galectin-3 resulted in smaller tumour size and fewer metastases in a co-implanted murine model of pancreatic cancer cells and pancreatic stellate cells (PSCs). Galectin-3 activates the integrin subunit beta 1 on PSCs, resulting in activated NF-κB through integrin-linked kinase, which influences the transcription of interleukin-8 [67]. The controversial role of galectin-3 in prognosis has been addressed in numerous studies, our present study indicated that galectin-3 showed limited prognostic value, with no direct correlation to OS and clinical characteristics in pancreatic cancer.

One reason for the conflict between the oncogenic mechanism and clinical features may be due to the function of galectins likely being dynamic during tumour progression, which is one part of the balance in the tumour microenvironment. Another reason could be that galectins might only play an important role in a certain group of patients, while a large population covering the small group could potentially lead to negative results. Differences in methodologies among these studies may have caused these controversial results; thus, standardization of the evaluation methods used for galectin expression and proper cut-off points are urgently needed. A consensus needs to be reached on research design and analysis of results in future studies, especially for other types of galectins that are less well studied. These findings provide hints for reconsidering the efficacy of galectin-targeting strategies, and identification of a specific population sensitive to galectins should be performed.

Although novel markers are being evaluated for more accurate prediction, a systematic review on serum tumor markers for the detection of recurrent pancreatic cancer reported that although the biomarker CA 19-9 has certain limitations, it remains the most widely used serum biomarker for postoperative surveillance of pancreatic cancer with a sensitivity and specificity 0.73 and 0.83, respectively [68]. Though galectins alone may not be an effective independent prognostic biomarker for pancreatic cancer compared to the performance of traditional clinical biomarkers such as CA19-9 and carcinoembryonic antigen (CEA) [69], the strategy to combine galectins with other biomarkers is worth consideration. A recent study has shown that the overexpression of galectin-3 and ezrin had stronger predictive value than either alone in cervical cancer [70]. In addition, another study in non-small cell lung cancer observed higher expression of cyclin D1 in galectin-3 free tumor tissues [71]. Similarly, to avoid the limitations of a single predictor, galectin-9 was included in a compelling immune biomarker panel to predict cancer-specific survival in pancreatic cancer, which might also benefit future prospective immunotherapy trials [28]. These results suggest that the potential role of galectins in predicting survival outcomes in cancer patients should not be underestimated and that the combination of biomarkers might be a more powerful prognostic tool.

The search for effective diagnostic serum markers of pancreatic cancer remains intense due to its relatively simple and noninvasive features. Various serum markers with potential diagnostic value have been widely investigated, particularly CA19-9, CA125 and CEA [41, 72]. CA 19-9 is still the most widely used diagnostic marker for pancreatic cancer due to its relatively high diagnostic accuracy, with an AUC reaching nearly 87% [73–75]. However, CA 19-9 is also elevated in some other conditions including other types of cancers as well as nonmalignant pathologies such as pancreatitis and cirrhosis. This has limited the sensitivity and specificity of CA19-9 for early detection. Furthermore, approximately 5–10% patients do not express CA19-9 [39]. Therefore, many studies have focused on the development of novel diagnostic panels to improve diagnostic accuracy based on this marker. Increased levels of circulating galectins have been reported in pancreatic cancer and some other cancer types, which has generated interest in galectins as potential diagnostic markers [76]. Galectin-1 and galectin-3 are intriguing markers for oral squamous cell carcinoma for the screening of higher risk populations [77]. The serum level of galectin-3 could assist as a diagnostic marker in bladder cancer patients [78]. With respect to diagnostic value in pancreatic cancer, our meta-analysis suggested that the pooled DOR of galectin-3 was 5.93, but the 95% CI was 0.96–36.72, indicating an unsatisfactory diagnostic accuracy and substantial heterogeneity. The most probable reason for the heterogeneous diagnostic performance of galectin-3 may be inconsistency in the control groups (healthy volunteer or pancreatitis patients). Some studies have proposed elevated circulating expression of galectin-3 as a potential biomarker for pancreatic cancer, and combined determination of galectin-3, CA19-9, and CA125 provided complementary diagnostic value for pancreatic cancer with a diagnostic sensitivity of 97.5% [22, 79]. However, those studies included a control group of healthy people and ignored patients with pancreatic or liver fibrosis despite the role of galectins in inflammation and collagen production [65]. Another study focused on the diagnostic value of galectin-3 in inflammatory pancreatic disease and indicated that galectin-3 is not an interesting biomarker for the detection of pancreatic adenocarcinoma [23]. Similarly, no significant difference in galectin-3 was observed between cirrhotic and hepatocellular carcinoma patients [80]. Therefore, although elevated galectin-3 has been observed in pancreatic cancer patients, galectin-3 alone might not be a viable diagnostic marker of pancreatic cancer due to its role in inflammatory diseases. Notably recent studies have suggested that the ligands of galectin-3 demonstrated relatively good performance for the diagnosis of cancer [81, 82]. Thus, adding galectin-binding glycoproteins in a galectin-based diagnostic panel might provide a strategy to improve the diagnostic performance of galectins. Hence, these results indicate the potential clinical diagnostic value of galectin-3, although more well-designed studies are needed to reach a definitive conclusion. Combination strategies are worthy of further exploration to improve the diagnostic capability of galectin-3.

Several limitations should be addressed for this meta-analysis. First, this meta-analysis includes a relatively small amount of studies with limited patients, which may have led to insufficient statistical power for analysing the diagnostic and prognostic role of galectins in pancreatic cancer. Second, given the lack of a standard cut-off value for galectins, different cut-off points were applied in the different included studies. Third, some of the HRs with 95% CIs were estimated by data extraction from the survival curves, which might convey certain statistical deviations. Fourth, we found that the different galectins, sample sizes, patient characteristics and cut-off values of the included studies might be potential sources of heterogeneity through subgroup analysis. Fifth, a potential publication bias and flawed methodologic design exists in the smaller studies included in the prognostic analysis. Finally, considering the limitations of the present study, additional well-designed studies with larger sample sizes need to be conducted.

## Conclusion

Our current research describes the first meta-analysis to comprehensively and systematically address the prognostic and diagnostic role of galectins in patients with pancreatic cancer. Our meta-analysis found that high expression galectin-1 and a low level of galectin-4 or galectin-9 were associated with worse prognosis, while galectin-3 expression did not show a correlation with prognosis and other clinical characteristics in pancreatic cancer patients. Although galectin-3 exhibited some diagnostic value in patients with pancreatic cancer in this meta-analysis, clinical application prospects remain to be validated.

## Supplementary information

**Additional file 1: Table S1.** Quality assessment of eligible studies with Newcastle–Ottawa Scale.

**Additional file 2: Fig. S1. a** Risk of bias and applicability concerns; **b** Risk of bias and applicability concerns. **Fig. S2. a** Diagnostic odds ratio of galectin-3 for diagnosis of pancreatic cancer; **b** Summary receiver operating characteristic (SROC) curve for the diagnostic accuracy of galectin-3 for the diagnosis of pancreatic cancer. **Fig. S3.** Funnel plot for publication bias. **a** Begg’s funnel plot; **b** Deeks’ funnel plot.

## Data Availability

The datasets used in this study are available from the corresponding author upon reasonable request.

## Abbreviations

AUC: area under the curve
CA125: carbohydrate antigen 125
CA15-3: carbohydrate antigen 15-3
CA19-9: carbohydrate antigen 19-9
CEA: carcinoembryonic antigen
CI: confidence interval
CRDs: carbohydrate-recognition domains
DFS: disease free survival
DOR: diagnostic odds ratio
FNs: false negatives
FPs: false positives
HR: hazard ratio
NLR: negative likelihood ratio
OR: odds ratio
OS: overall survival
PLR: positive likelihood ratio
PSCs: pancreatic stellate cells
SROC: summary receiver operating characteristic curve
TNM: tumor node metastasis
TNs: true negatives
TPs: true positives
