# Supplementary material for: Prognostic and diagnostic significance of galectins in pancreatic cancer: a systematic review and meta-analysis
Source: Cancer Cell Int. 2019 Nov 21;19:309. doi: 10.1186/s12935-019-1025-5 (PMC6873495; doi:10.1186/s12935-019-1025-5)
Supplement: Supplementary file 1 — Additional file 1: Table S1. Quality assessment of eligible studies with Newcastle–Ottawa Scale. [file 12935_2019_1025_MOESM1_ESM.docx]

**Additional file 1: Table S1 Quality assessment of eligible studies with Newcastle-Ottawa Scale**

| **Study** | **NOS** | **Selection** | **Comparability** | **Outcome** |
| --- | --- | --- | --- | --- |
| Chen2012 [16] | 6 | ★★★^*^ | ★^*^ | ★★^*^ |
| Tang2015 [14] | 7 | ★★★ | ★★ | ★★ |
| Shimamura2002 [17] | 8 | ★★★ | ★★ | ★★★ |
| Gaida2011 [18] | 6 | ★★★ | ★^*^ | ★★^*^ |
| Shimura2017 [21] | 8 | ★★★^*^ | ★★^*^ | ★★★ |
| Maftouh2014 [23] | 6 | ★★★^*^ | ★★ | ★^*^ |
| Hu2019 [24] | 8 | ★★★ | ★★ | ★★★ |
| Sideras2017 [25] | 7 | ★★★ | ★^*^ | ★★★ |

^*^ The score was produced by discussion.
