# Supplementary material for: Prognostic and diagnostic significance of galectins in pancreatic cancer: a systematic review and meta-analysis
Source: Cancer Cell Int. 2019 Nov 21;19:309. doi: 10.1186/s12935-019-1025-5 (PMC6873495; doi:10.1186/s12935-019-1025-5)
Supplement: Supplementary file 2 — Additional file 2: Fig. S1. a Risk of bias and applicability concerns; b Risk of bias and applicability concerns. Fig. S2. a Diagnostic odds ratio of galectin-3 for diagnosis of pancreatic cancer; b Summary receiver operating characteristic (SROC) curve for the diagnostic accuracy of galectin-3 for the diagnosis of pancreatic cancer. Fig. S3. Funnel plot for publication bias. a Begg’s funnel plot; b Deeks’ funnel plot. [file 12935_2019_1025_MOESM2_ESM.pdf]

a

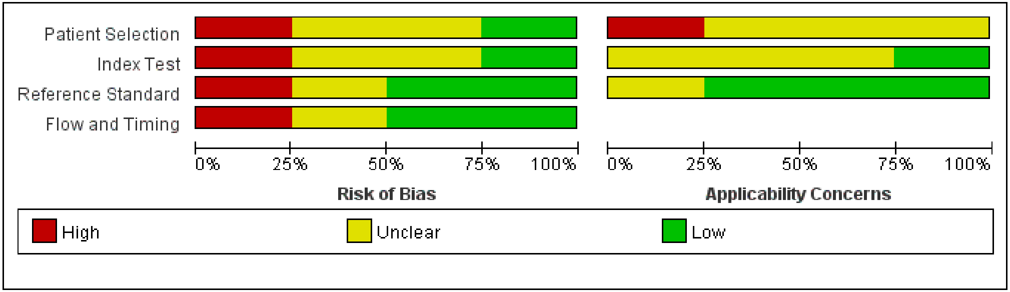

b

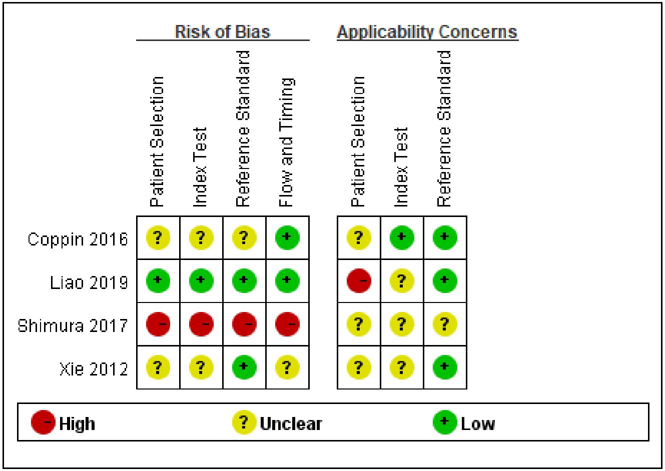

Fig. S1 a Risk of bias and applicability concerns; **b** Risk of bias and applicability concerns.

a b

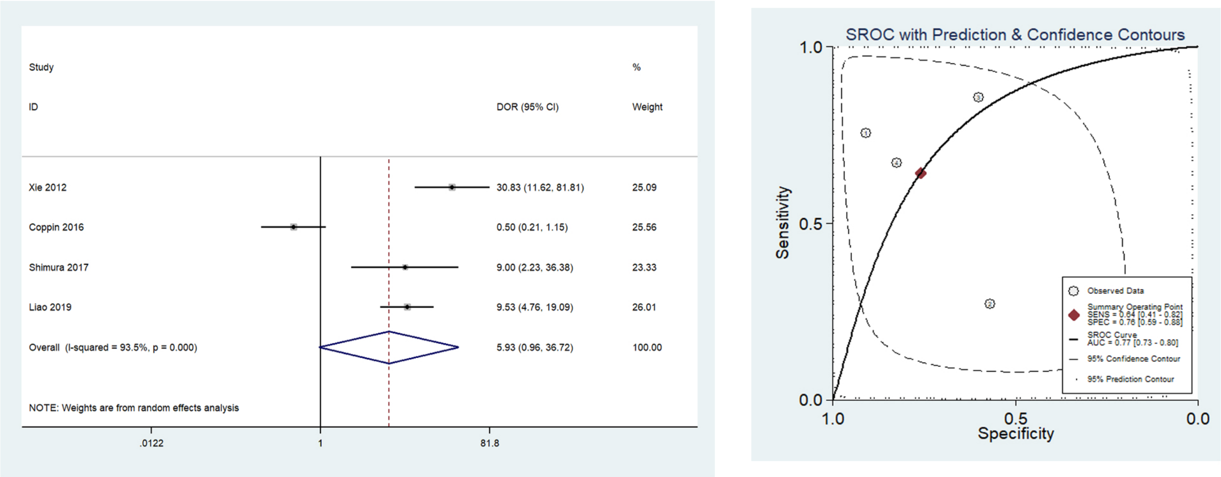

Fig. S2 a Diagnostic odds ratio of galectin-3 for diagnosis of pancreatic cancer; **b** Summary receiver operating characteristic (SROC) curve for the diagnostic accuracy of galectin-3 in diagnosing for pancreatic cancer.

a **b**

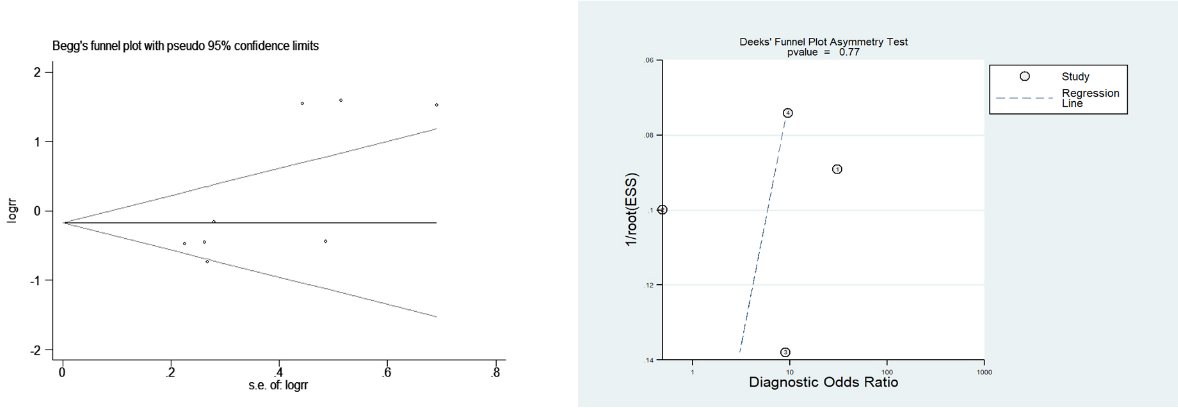

Fig. S3 Funnel plot for publication bias. **a** Begg's funnel plot; **b** Deeks' funnel plot.
